# Association between Dental Caries and Obesity in Children and Young People: A Narrative Review

**DOI:** 10.1155/2019/9105759

**Published:** 2019-05-02

**Authors:** Abdulmonem A. Alshihri, Helen J. Rogers, Mohammed A. Alqahtani, Mohammed S. Aldossary

**Affiliations:** ^1^Department of Prosthodontics and Biomaterials, College of Dentistry, King Saud University, Riyadh, Saudi Arabia; ^2^Division of Pediatric Dentistry, School of Clinical Dentistry, University of Sheffield, Sheffield, UK; ^3^Division of Pediatric Dentistry, Department of Dentistry, Ministry of Health, Riyadh, Saudi Arabia

## Abstract

**Objectives:**

To explore the association between obesity and dental caries in children and adolescents. Furthermore, to consider the possible reasons behind this relationship.

**Methods:**

A database search for papers published between January 2015 and May 2018, inclusive, addressing the association between obesity and dental caries was conducted. A review and critical appraisal of all included studies was performed.

**Results:**

Twenty-six studies were included in this review from different populations worldwide. Eight studies assessed the primary dentition, nine studies were conducted on permanent dentition, and remaining nine studies on both dentitions. The results regarding the association between obesity and dental caries were conflicting and inconsistent. Nine studies concluded that there was no relationship between obesity and dental caries. A positive association was reported in five studies, while the inverse association was reported in eleven studies. Studies included in this review had significant variations in methodology and the investigated cofactors. Possible explanations of the controversial association between obesity and dental caries are discussed in this review.

**Conclusion:**

Both obesity and dental caries are multifactorial diseases, and their association is far more complex that can be explained by a single common risk factor, presenting evidence for the complexity of this association.

## 1. Introduction

Obesity and dental caries are growing public health problems worldwide. Both obesity and dental caries are considered to be chronic, highly prevalent, and multifactorial conditions, with significant and potentially lifelong impacts on the lives of children and young people [1–4]. The two conditions are thought to share common contributing factors, including biological, genetic, socioeconomic, cultural, dietary, environmental, and lifestyle factors [5, 6].

For this reason, a relationship between obesity and dental caries seems logical. Further knowledge of this relationship could enable the development of more effective and efficient targeted public health initiatives to reduce the prevalence of both obesity and dental caries [6–8].

Numerous studies have investigated the association between obesity and dental caries in different countries, and in both primary and permanent dentitions [2, 9]. However, the results have always been controversial and inconclusive. The data are inconsistent regarding the existence of a relationship and the nature and direction of the association. Some studies found no association, whereas others reported a positive correlation or an inverse relationship between the two conditions [10].

Few systematic reviews of the literature regarding the association between obesity and dental caries were published in 2012-2013 [2, 9, 10]. There was agreement between all of these systematic reviews regarding the inconclusive literature and the need for further analysis of this association and its confounding variables.

A more recent systematic review in 2015 included only longitudinal studies and concluded that the evidence of the association between obesity and dental caries was conflicting and remains inconclusive. These inconsistent results are influenced by discrepancies in assessments, setting, and measurements [11].

Since the publication of these reviews, many further studies have been conducted to assess the association between obesity and dental caries in various populations and countries [12–16].

This narrative review was conducted to investigate the reported association between obesity and dental caries in children and adolescents in the most recent publications. In addition, this review aimed to provide a narrative synthesis of the possible explanations and underlying causes of the association between obesity and dental caries.

## 2. Materials and Methods

A search of PubMed/Medline, ScienceDirect, Scopus, and Google scholar databases was conducted for papers published between January 2015 and May 2018, inclusive. Search terms surrounding “children” and “adolescents” were combined with terms relating to “caries,” “body mass index,” “obesity,” and “weight.” This search was supplemented by manual searching of reference lists from each relevant paper identified.

All English language studies addressing the association between obesity and dental caries in children and adolescents were included. Studies relating to prevalence and risk factors only were excluded, as were studies involving participants over the age of 18 years. A review of the associations reported in these studies was conducted, with a synthesis of the potential explanations.

## 3. Results

A total of 26 studies of various global populations were included in this review. The summary of these studies is presented in Table 1.

Amongst the included studies, eight assessed the primary dentition, nine investigated the permanent dentition, and the remaining nine studies investigated both dentitions. The results were varied, with nine studies concluding that there was no relationship between obesity and dental caries. A positive association between the two conditions was reported in five studies, whilst an inverse association was identified by eleven studies. Importantly, only one study found that prevalence of dental caries was higher in both obese and underweight children, showing a positive association in both directions.

Most of the studies used a cross-sectional design (*n* = 22). Only three studies were longitudinal and, interestingly, all of these three studies were conducted on permanent dentition and concluded a positive association. One study utilized a case-control design with children in the primary dentition and identified a positive association.

## 4. Discussion

The studies included in this review identified varying associations between caries and obesity in children and young people. These associations and potential explanations for them are explored further below.

### 4.1. Primary versus Permanent Dentitions

This review found no convincing evidence to demonstrate a specific association between obesity and dental caries in the primary dentition when compared to the permanent dentition. Despite the limited evidence, it appears that more studies tend to show that young children with dental caries in the primary dentition are underweight compared to children without caries, which is an inverse association [14, 18–20]. Obese older children and adolescents are more likely to have dental caries in their permanent teeth, which is a positive association [33–35]. However, contradictory conclusions were reported in other investigations (Table 1).

### 4.2. Positive Association

Multiple studies within this review have demonstrated that both obesity and caries share common risk factors, which would support a positive association. Drawing from these studies and the wider literature, the possible explanations for this positive association are considered below.

#### 4.2.1. Role of Diet

The role of diet is significant in the development of obesity and dental caries. Both conditions share some common diet-related risk, which influences the incidence of both obesity and dental caries. These diet factors include poor food choices, dietary habits, frequency and high consumption of fermentable carbohydrates, consumption of sweetened junk foods, and high-calorie and cariogenic diets [13, 30].

Some studies emphasize that frequent and excessive intake of fermentable sugars is the critical common predisposing factor for obesity and dental caries [24, 34].

#### 4.2.2. Biological Indicators

It has been suggested within the literature that obesity may alter body homeostasis, which in turn may result in an increase in dental caries [40]. Therefore, the association between different biological indicators of obesity and dental caries has been investigated.

Lower stimulated secretion rates of saliva, higher concentrations of secretory immunoglobulin A (sIgA), and different oral microbial profiles were reported in obese individuals [41]. Some authors suggest that obesity may lead to changes in concentrations of free sialic acid, total protein, and phosphate as well as peroxidase activity in stimulated saliva, which may promote dental caries [41]. However, it was not possible to confirm whether the association is due to systemic changes or other possible factors such as diet and oral hygiene habits [40, 41].

#### 4.2.3. Role of Lifestyle

The literature has also suggested that positive associations between obesity and dental caries may be the result of other shared contributing factors, such as those relating to lifestyle.

Lifestyle characteristics that may play a role in the development of both conditions include reduced physical activity, increased consumption of snacks, and increased time spent watching TV and using new multimedia technologies [24, 42].

Additionally, it has been suggested that both conditions are more prevalent in some specific communities due to unhealthy food, lower parental education levels, and inability to obtain sufficient health care and services [13, 26, 43].

### 4.3. Inverse Association

In contrast to the studies reporting a positive association between obesity and dental caries, a number of studies showed an inverse association where increased prevalence of caries was associated with being underweight (Table 1). Some theories may explain this inverse relationship.

#### 4.3.1. Role of Diet

Although sugar is one accepted risk factor for obesity and dental caries, the inverse relationship may also be attributable to dietary patterns. Obese children and adolescents might consume more fatty foods, fried foods, and unrefined carbohydrates, but not necessarily more foods high in sugar and refined carbohydrates. This could increase obesity, but not necessarily have a direct link to dental caries [36, 44, 45].

Further to the diet itself, the process of mastication has also been reported to be affected by dental caries, which in turn could lead to reduced nutritional intake by children and young people [46]. Gilchrist et al. [3] reported that some children with caries may have restricted diets for lengthy periods of time, relating to difficulty eating hard foods, and getting food stuck in their teeth.

The effects of these dietary limitations may extend further than just weight. A number of earlier studies have investigated the association between iron-deficiency anemia, a common form of malnutrition, and dental caries [47]. Rodd and Blankenstein identified a statistically significant increase in the number of teeth which required extraction amongst UK children with anemia, compared to those without, indicating that caries severity may be greater in anemic children [48].

Further to this, dietary nutrients such as vitamins A and D, calcium, and phosphate play important roles in tooth morphology, chemical composition, and tooth eruption patterns. Reduced consumption of these nutrients may in turn affect the susceptibility of teeth to dental caries [19, 37, 49].

#### 4.3.2. Untreated Caries and Consequences

Another explanation for the inverse association might be that children with untreated caries could experience pain and infection, thus preventing them from consuming adequate nutrition. In addition, other factors that contribute to overall wellbeing could be affected, including the ability to sleep, which in turn may lead to malnutrition and growth impairment [12, 18, 20, 25, 36]. The wider literature suggests that underweight children gained weight after receiving dental treatment [19, 50].

However, this possible explanation is more apparent in populations with a high proportion of severe and untreated dental caries [28, 37].

#### 4.3.3. Saliva Production

Another possible explanation for an inverse relationship is that saliva production increases due to increased food consumption in obese groups [36]. The protective effect of saliva as a mechanical cleanser and pH buffer could thus reduce the incidence of dental caries [36, 51].

#### 4.3.4. Socioeconomic Status

Many authors have suggested that both being underweight and having dental caries could be due to poverty and low socioeconomic status [15, 16, 18, 19, 25, 28, 37, 38]. Nonetheless, this association is not present in all populations, and there is great variation globally that can be partially attributed to cultural differences [52].

### 4.4. No Relationship

Some investigators found no correlation between obesity and dental caries. One possible explanation for this stems from the fact that both obesity and dental caries are multifactorial in etiology, and various genetic and environmental factors have an impact on them. Consequently, the many confounding factors, including age, gender, and lifestyle, might determine the development of these conditions [31, 32]. Nonetheless, the literature suggests that dietary factors, oral hygiene practices, and socioeconomic status are more significant risk factors for dental caries than for the development of obesity [16, 22].

Moreover, as stated previously, obesity can be due to an increased intake of dietary fats, which has less influence on the development of dental caries than a diet high in sugar [36, 44, 45].

Interestingly, where proper oral hygiene is maintained with adequate fluoride exposure, dental caries prevalence has decreased despite increases in sugar consumption [21].

### 4.5. Methodological Considerations

This section will discuss the possible methodological explanations for the variation in relationships observed in the literature.

#### 4.5.1. Age of Participants

The differences between the reports might be related to diversity in the age of study subjects. While some studies investigated the association between obesity and dental caries in narrow or wide age ranges, one study investigated caries in children in a single age group (8 years old) [28]. The widest age range among the included studies was noted in da Silva et al. who included 3- to 15-year-old participants [30].

It is possible that caries is an age-related, cumulative condition, and thus, older groups are more likely to exhibit higher prevalence of dental caries [19]. However, in younger children, the prevalence of dental caries may decrease with increasing age as a result of the exfoliation of primary teeth [20].

One important consideration of studies incorporating a wide range of age groups is that children of different ages may have particularly varied dietary habits and lifestyles [20]. Children and adolescents become more independent of food choice with increasing age, which is a significant influence in regard to this association. Older children who are overweight or obese often have dietary lifestyles involving frequent eating and are therefore more likely to experience dental caries as a result [49].

#### 4.5.2. Gender of Participants

Gender differences may contribute to differences in diet, eating patterns, and physical activity, as well as in time of tooth eruption [49]. Moreover, gender-related differences in the amount of body fat result from differences in growth milestones, body structure, and hormonal effects [49]. These may influence the prevalence of obesity and dental caries, and thus their correlation [21].

The majority of the included studies attempted to maintain a consistent distribution of male and female participants in their samples. However, Bhayat et al. [36], and Alghamdi and Almahdy [38], studied the association between obesity and dental caries in a sample comprised only of boys.

While some studies reported more dental caries in boys [19, 20], Quadri et al. [12] found more dental caries in girls. By contrast, some studies concluded that there is no difference in dental caries between both genders [18, 25].

With regard to obesity, girls were more likely to be obese [18, 25, 26]. Thus, similar gender distribution is an important factor to consider avoiding misleading conclusions [35].

#### 4.5.3. Sample Size

Sample sizes were clearly disparate between the included studies. The sample sizes of the 26 studies ranged from 100 to 32461, and the median sample size was 521. The majority of studies included less than 1,000 participants (*n* = 20).

Increasing the sample size was recommended by most of the studies, particularly to overcome the effect of dividing the body mass index (BMI) scores into subgroups.

#### 4.5.4. Dental Caries Diagnoses

The technique for diagnosis of dental caries diagnosis used in all included studies was direct visual oral examination with no radiograph. This detection technique typically results in an underestimation of dental caries prevalence [24].

Another concern relates to the caries indices used in these studies and the different diagnostic criteria employed.

Most studies used decayed, missing due to caries, filled teeth indices for primary and permanent dentitions (dmft/DMFT) according to the World Health Organization (WHO) criteria [12, 16, 19, 20]. According to the WHO criteria, only the cavitation is inspected and recorded as a carious lesion, and noncavitation carious lesions are not included.

One study used both DMFT and decayed, missing due to caries, filled, surfaces (DMFS) [35]. By contrast, Qomsan et al. [24] reported the DFT of the permanent dentition, and Aluckal et al. measured the (dft) of the primary dentition [23]. Both studies did not include the missing teeth due to caries (mt/MT), which would alter their results.

The American Academy of Pediatric Dentistry criteria were used to record severe early childhood caries in children that participated in a study by Davidson et al. [17]. These criteria include noncavitation lesions in the definition of dental caries in young children.

The International Caries Detection and Assessment System (ICDAS-II) is used to examine cavitation caries and early enamel caries and was employed by some of the included studies [14, 32].

The National Institute of Dental Research (NIDR) criteria employed by Farsi et al. [25] and Elkhodary et al. [18] is an old caries scoring system, which was last updated in 1991 and is considered an intentionally conservative system, with only clear cavitation being recorded as a carious lesion [53].

Studies using different diagnostic criteria could result in different caries prevalence rates, which would affect the results and potential relationship with obesity. The impact of this variation in reporting clinical outcomes amongst researchers has not gone unnoticed, having been highlighted by authors of systematic reviews previously [54, 55]. This has led to the development of the Outcomes in Trials for Management of Caries Lesions (OuTMaC) study. Whilst this study is ongoing, it aims to develop a core outcome set for trials investigating clinical management of caries lesions in primary or permanent teeth [27].

The analyzed dentition varied between studies regardless of the target age, especially in the mixed dentition ages. The inclusion of both primary and permanent dentitions at different ages may have skewed the caries prevalence and affected any association between obesity and dental caries [36].

#### 4.5.5. Anthropometric Measures

The included studies used a variety of methods for assessing obesity and anthropometric measures. The majority of studies assessed the obesity based on BMI and did not report other anthropometric outcomes. In addition, different BMI indices and growth references were applied in these studies [13, 19, 21, 24, 36, 38].

Some studies relied on the recommended age- and gender-specific WHO growth references, which were expressed as *z*-scores and categorized into four subgroups: underweight, normal, overweight, and obese [22, 29, 36, 38, 39].

The BMI for age and gender percentiles according to the Centers for Disease Control and Prevention (CDC) was employed by some of the studies [12, 16, 19, 23, 24, 28]. Percentiles are derived from corresponding age- and gender-adjusted *z*-scores and categorized into four subgroups as underweight, normal, risk of overweight, and overweight.

By contrast, other studies employed the international BMI recommended by the World Obesity/Policy and Prevention (formerly International Obesity Task Force; IOTF), which uses only two categories, “not overweight” and “overweight” [13, 21]. The age- and gender-specific international BMI criteria (iso-BMI) are based on IOTF guidelines and have four categories similar to the WHO criteria. These criteria were applied by Qadri et al. [34].

Davidson et al. [17] used both WHO and CDC criteria, and Liang et al. [20] applied all the criteria from all three (WHO, CDC, and IOTF).

The different references that were applied in these studies might have altered the findings and comparisons between their results should be made with caution. Interestingly, a systematic review showed a significant association between obesity and dental caries when the BMI for age and gender percentile (CDC) were reported and no significant associations when *z*-scores (WHO) were reported [2].

The BMI thresholds in the included studies were based on different growth and development charts between different countries. For instance, Liang et al. [20] used a Chinese chart, Farsi et al. [25] used a Saudi chart, and Kumar et al. [15] used an Indian growth chart. Consequently, the different classification criteria produce different groups. Furthermore, the methodology for BMI grouping and distribution in the studies was inconsistent. It is therefore recommended to distribute samples into the full range of BMI categories to maintain a normal distribution [9, 17].

In contrast, Americh-Torres et al. [32] grouped the participants into three categories (normal weight, overweight, and obese), whilst Soares et al. [14] combined the overweight and obese groups together, which resulted in three groups: underweight, normal, and overweight/obese.

Although BMI is clearly an effective screening tool, there is growing concern regarding the accuracy of using BMI to precisely detect obesity. BMI is calculated using height and weight, which vary widely during growth, especially for children [53, 56]. Additionally, BMI has comparative limitations due to inherent differences in body fat percentage between males and females [17].

Due to the limitations of BMI, some studies have evaluated obesity using other diagnostic techniques or in combination with them.

Waist circumferences were reported in three of the included studies as a supplement to the BMI measured according to CDC percentiles [18, 22, 25]. In addition to BMI, Li et al. [33] recorded waist circumference, waist-to-hip ratio, waist-to-height ratio, and skinfold thickness. These techniques are more precise, accurate, and reliable tools for defining obesity levels; yet, they are not widely used within the literature [24, 25, 57].

#### 4.5.6. Statistical Analyses

There were different statistics used in the included studies using correlation and regression to investigate the association between obesity and dental caries. Some studies used Pearson's correlation [23, 28], while others used Spearman's correlation [18, 24, 29]. Multiple linear regression was employed by some of the studies [14, 17, 21, 36], while others used Poisson's regression [19, 22, 34, 39], or logistic regression models [12, 20, 21, 30–33].

Careful consideration and selection of statistical tests are necessary to reach reliable conclusions. Moreover, both clinical significance and statistical significance should be interpreted carefully. The results of two studies [15, 16] showed an inverse association between obesity and dental caries, which was not statistically significant, thus reaching a conclusion of no association. In contrast, Kottayi et al. [13] reported a nonsignificant positive association between both conditions, leading to a conclusion of no association.

#### 4.5.7. Confounding Factors

The studies included within this review were conducted in a range of developed and developing countries. Different populations and communities have varied political structures, and underlying cultural influences have been shown to hold an important role in caries prevalence [52]. Furthermore, the variation in dental and health services and facilities, including proper health education and dietary counselling, can affect oral and general health outcomes [21, 30]. The samples used in some studies included children and adolescents from different social and ethnic backgrounds; yet, these be representative of the source populations.

Additionally, wide variation was noted between studies regarding the assessment and control of potential confounding factors. These factors relate to age, gender, lifestyle, dietary habits, oral hygiene, socioeconomic status, race/ethnicity, physical activity levels, and even types of schools. Each study controlled for a few of these confounding factors, but other potential confounding factors were not assessed. All of these uncontrolled confounders could have biased the results [20, 25, 36].

The challenge in exploring the association lies in determining the impact of potential confounding factors. Careful consideration should be given to future studies in this remit to ensure that the study design minimizes any impact from these important confounding factors.

#### 4.5.8. Study Design

The inconsistent associations reported between obesity and dental caries could be due to methodological limitations and variations in study designs.

Clearly, for ethical reasons, any randomized controlled trials in this field would be precluded. As a result, the majority of the included studies were cross sectional. The main limitation of cross-sectional studies is that definitive information about cause and effect relationships cannot be determined. These types of study cannot identify risk factors and often miss many of the confounding factors that influence a particular problem over time when studying chronic diseases such as obesity and dental caries. Determining the weight and impact of a confounding factor in an observational study can be difficult [19, 20].

Further longitudinal studies could provide greater understanding of the cause, mechanism, and consequences of any possible association between these conditions [20, 39].

Longitudinal studies would benefit from adequate adjustment for confounding variables and from the use of continuous outcomes. This could be more effective for the development of more realistic models for predicting such chronic conditions.

The studies identified in this narrative review confirm the range in associations between caries and obesity in children and young people that have been highlighted in previous systematic reviews [2, 9–11]. Furthermore, this review provides a detailed consideration of the potential reasons behind these varied associations and highlights the complexity of this relationship.

This study focused on the more recent published literature in this field, in order to gain more contemporary findings relating to the associations between the two conditions, yet this could be considered a limitation of this study. Furthermore the range of databases searched was not extensive, though this is generally acceptable for a narrative review of the subject.

Future research in this area should be directed to the conduct of high-quality longitudinal studies, designed to minimize the impacts of confounding variables. A closer analysis of the associations between caries and obesity in children and young people in relation to differing populations and cultural values could also contribute greatly to the evidence base in this field.

## 5. Conclusion

Both obesity and dental caries are multifactorial conditions, and it is difficult to assess all of the associated risk factors simultaneously. The association is far more complex than can be explained by a single common risk factor or dietary habits alone.

The presence of multiple confounding factors resulted in an inability to draw a firm conclusion regarding the association. This review provides additional evidence for the complexity of this association.

## Figures and Tables

**Table 1:** Summary of the included studies.

Investigated dentition	Association between obesity and dental caries. Author (country, sample size, age in years)			
	Positive	Inverse	No relationship	Positive in two directions
Primary	Davidson et al. [17] (Canada, 235, 2 to 6)^*∗*^	Soares et al. [14] (Brazil, 285, 3 to 5) Elkhodary et al. [18] (KSA, 820, 3 to 6) Bafti et al. [19] (Iran, 1482, 3 to 6) Liang et al. [20] (China, 32461, 7 to 9)	de jong-lenters et al. [21] (Netherlands, 230, 5 to 8) Paisi et al. [22] (UK, 347, 4 to 6)	Aluckal et al. [23] (India, 433, 2 to 6)

Primary and permanent	Qomsan et al. [24] (KSA, 386, 6 to 12)	Quadri et al. [12] (KSA, 360, 6 to 15) Farsi et al. [25] and Schwendicke et al. [26, 27] (KSA, 915, 7 to 10) Guo et al. [28] (China, 744, 8 to 8.5)	Mitrakul et al. [25] (Thailand, 100, 6 to 12) da Silva et al. [30] (Brazil, 237, 3 to 15) Araujo et al. [31] (Brazil, 313, 8 to 10) Münevveroğlu et al. [16] (Turkey, 856, 6 to 12) Almerich-Torres et al. [32] (Spain, 1326, 6/12/15)	—

Permanent	Li et al. [33] (Hong Kong, 282, 12/15/18)^*∗∗*^ Qadri et al. [34] (Germany, 694, 9 to 12)^*∗∗*^ Basha et al. [35] (India, 785, 13 +3 years)^*∗∗*^	Bhayat et al. [36] (KSA, 402, 12 to 14) Chauhan et al. [37] (India, 275, 6 to 15) Alghamdi and Almahdy et al. [38] (KSA, 610, 14 to 16) Fernández et al. [39] (Brazil, 1210, 8 to 12)	Kottayi et al. [13] (India, 2000, 12 to 15) Kumar et al. [15] (India, 1092, 11 to 14)	—

^*∗*^Case-control study. ^*∗∗*^Longitudinal study. The rest are cross-sectional studies.
